# Transcriptome profiling reveals that foliar water uptake occurs with C_3_ and crassulacean acid metabolism facultative photosynthesis in *Tamarix ramosissima* under extreme drought

**DOI:** 10.1093/aobpla/plab060

**Published:** 2022-01-17

**Authors:** Xia Yan, Yan Chang, Weijia Zhao, Chaoju Qian, Xiaoyue Yin, Xingke Fan, Xinyu Zhu, Xiangqiang Zhao, Xiao-Fei Ma

**Affiliations:** 1 School of Life Sciences, Nantong University, Nantong 226019, Jiangsu, China; 2 Key Laboratory of Inland River Ecohydrology, Northwest Institute of Eco-Environment and Resources, Chinese Academy of Sciences, Lanzhou 730000, Gansu, China; 3 Key Laboratory of Stress Physiology and Ecology in Northwest Institute of Eco-Environment and Resources, Chinese Academy of Sciences, Lanzhou 730000, Gansu, China; 4 College of Resources and Environment, University of Chinese Academy of Sciences, Beijing 100049, China

**Keywords:** Carbon assimilation, foliar water uptake, gene expression, *Tamarix ramosissima*, transcriptome profiling

## Abstract

*Tamarix ramosissima* is a typical desert plant species that is widely distributed in the desert areas of Northwest China. It plays a significant role in sand fixation and soil water conservation. In particular, how it uses water to survive in the desert plays an important role in plant growth and ecosystem function. Previous studies have revealed that *T. ramosissima* can alleviate drought by absorbing water from its leaves under extreme drought conditions. To date, there is no clear molecular regulation mechanism to explain foliar water uptake (FWU). In the present study, we correlated diurnal meteorological data, sap flow and photosynthetic parameters to determine the physical and biological characteristics of FWU. Our results suggested that the lesser the groundwater, the easier it is for *T. ramosissima* to absorb water via the leaves. Gene ontology annotation and Kyoto Encyclopaedia of Genes and Genomes pathway analysis of the transcriptome profile of plants subjected to high humidity suggested that FWU was highly correlated to carbohydrate metabolism, energy transfer, pyruvate metabolism, hormone signal transduction and plant–pathogen interaction. Interestingly, as a C_3_ plant, genes such as *PEPC*, *PPDK*, *MDH* and RuBP, which are involved in crassulacean acid metabolism (CAM) photosynthesis, were highly upregulated and accompanied by FWU. Therefore, we proposed that in the case of sufficient water supply, C_3_ photosynthesis is used in *T. ramosissima*, whereas in cases of extreme drought, starch is degraded to provide CO_2_ for CAM photosynthesis to make full use of the water obtained via FWU and the water that was transported or stored to assimilating branches and stems. This study may provide not only an important theoretical foundation for FWU and conversion from C_3_ plants to CAM plants but also for engineering improved photosynthesis in high-yield drought-tolerant plants and mitigation of climate change-driven drought.

## Teaser

Under extreme drought, desert plants can not only absorb underground water through their roots, but also resist drought from foliar water uptake. In this study, based on RNA-seq analysis together with moisture treatment, we found there existed a significant shift in the carbon assimilation pathway during foliar water uptake. This work supports the ecological significance of energy transmission of desert plants to cope with water deficit.

## Introduction

Bidirectional water transport models have gradually been accepted in plants (Goldsmith *et al.* 2013), instead of the traditional soil–plant–atmosphere continuum (SPAC; Philip 1966) model. Under this new model, water could be absorbed from leaves (foliar water uptake, FWU) in the form of dew (Liu *et al.* 2020), fog (Wang *et al.* 2019), clouds (Eller *et al.* 2015), light rain showers that only wet the leaves and high humidity (Gong *et al.* 2019). Foliar water uptake is assumed to be a significant source contributing to the water balance of terrestrial ecosystems as well as water from roots in the form of precipitation occurring as rain or snow (Limm *et al.* 2009). With people’s understanding of aggregation of desertification under global climate change, it is becoming increasingly important to maintain the stability of fragile ecosystems (Berry *et al.* 2019). Many studies have shown that the amount of water from foliar absorption increases with the aggregation of drought. In the coastal prairie ecosystem of California, 28–66 % of the water intake relies on FWU (Corbin *et al.* 2005). This percentage increases to 34 % in the redwood tree ecosystem (Dawson and Goldsmith 2018) and reaches 74 % in the Negev desert, Israel (Kidron 2010). To date, at least 233 species spanning 77 plant families and six major biomes have demonstrated some capacity for FWU (Binks *et al.* 2020).

Previous studies have extensively focused on the understanding of when, where and how FWU occurs (Eller *et al.* 2015). Most studies support that FWU occurs across stomata (Boanares *et al.* 2019). However, FWU occurs during periods when stomata are mostly closed (i.e. at night). Therefore, it is considered that specialized structures such as cuticles, trichomes, hydathodes or scales (Berry *et al.* 2019; Schreel *et al.* 2020) are also important for the water to enter the plant to support FWU. The water from FWU is routinely incorporated into plant vascular networks, which is detailed in SPAC: (i) entry into the mesophyll and use for photosynthesis or capacitance (Eller *et al.* 2015), and incorporation into photosynthetic pathways (Lehmann *et al.* 2018); (ii) entry into the vasculature (Berry *et al.* 2019), as well as transportation to the phloem and possibly the xylem (Yan *et al.* 2015); (iii) transpiration back into the atmosphere. However, there is no clear explanation for species-level variation in FWU, and the molecular regulation network of FWU is seldom addressed.

Across C_3_ to C_4_ plants, the circadian clock regulates the exchange of water vapour and carbon dioxide between leaves and the atmosphere through stomatal conductance and photosynthesis. On the other hand, night-time starch mobilization could also be regulated by the circadian clock to maintain a steady supply of carbon until dawn (Smith and Zeeman 2020). Therefore, there must be some molecular regulation between starch synthesis and water usage. In fact, the C_3_ and C_4_ or the C_3_ and crassulacean acid metabolism (CAM) pathways coexist in many plants (Svensson *et al.* 2003). Overexpression of essential C_4_-photosynthetic genes such as *PEPC*, *MaeB* and *NADP-ME* in C_3_ plants such as *Arabidopsis*, tobacco, rice, wheat and potato not only improved photosynthesis but also improved the tolerance to various environmental stresses, especially drought (Yadav *et al.* 2020). It is widely known that CAM photosynthesis can coexist in leaves that exhibit any of the other types of photosynthesis known in terrestrial plants (i.e. C_3_, C_4_ and C_3_–C_4_ intermediate) (Winter *et al.* 2019), and C_3_ and CAM metabolism can even both exist within one leaf (Yuan *et al.* 2012). Thus, it is reasonable to infer that desert plants exhibit reduced photorespiration and improved water use efficiency with C_3_ and CAM intermediate photosynthesis (Monson 1989). In desert plants, genes that have a higher expression at night and a lower expression in the daytime may be important for efficient carbon fixation and water storage (Zhang *et al.* 2019). For example, some aquaporins (which would adjust the flow of water in mesophyll cells, a bundle sheath and phloem or possibly the xylem; Mayr *et al.* 2014), were differently expressed to regulate the transport of moisture in the air from assimilating branches to the secondary branches and the trunk stems but not to the taproot xylem or the soil (Yan *et al.* 2015). All those genes should be linked to circadian clock regulation, stomatal movement, sugar metabolism and transport pathways (Yang *et al.* 2017). Proteins involved in osmotic adjustment, ion transport, energy metabolism and light response may play important roles in the C_3_ to CAM transition (Guan *et al.* 2021). These processes provided important insights into the CAM transition and may facilitate plant growth in arid environments (Heyduk *et al.* 2019) and help enhance crop resilience for global food security (Guan *et al.* 2021).

*Tamarix ramosissima* is a desert forest tree species that is widely distributed in drought-stricken areas that have an annual precipitation of <200 mm in North China. Based on the δ ^13^C value, which is often used to distinguish carbon assimilation or water use strategy, *T. ramosissima* is traditionally regarded as a C_3_ plant (Zhang *et al.* 2017). However, variations in the δ ^13^C value caused by the influence of temperature, wind speed, precipitation and relative humidity (RH) (Zhang *et al.* 2018) also reflect conversion of water use strategy. In addition, a complete set of core genes of C_4_ carbon fixation were found not only in the two *Tamarix* species, namely, *T. ramosissima* and *Tamarix chinensis* (Swaminathan *et al.* 2020), but also in *Reaumuria soongorica*, which belongs to the same family as *Tamarix* spp. according to transcriptome profiling data (Shi *et al.* 2013). The presence of these genes enables the activation of the CAM pathway at night for C_3_ plants, but the molecular process is not yet validated.

In C_3_ plants, if leaf-wetting occurs during the night-time, photosynthesis will not be affected to the same extent as if the wetting events were to occur during the day. Compared with C_3_ plants if leaf-wetting was to occur at night in CAM plants, we would expect productivity to increase. These contrasting cases highlight the potential trade-off between carbon fixation and increased water availability, in which we might expect a certain optimum in terms of cumulative FWU and the timing of wetting events that may impact the types of traits that influence the ability and extent of FWU (Dawson and Goldsmith 2018). Thus, we proposed that CAM photosynthesis is activated in *Tamaricaceae* plants to improve water usage efficiency under extreme drought environments by moving the stomatal opening time and primary CO_2_ uptake and fixation events to night-time. To test this hypothesis, RNA-seq transcriptome profiling was performed to identify the differently expressed genes in plants under conditions of high moisture and natural fields at night. The differential gene expression will reveal not only the mechanism of FWU but also the mechanism underlying the shift in carbon fixation under drought. This study represents a particularly exciting frontier with both basic and applied implications for plant–water relations.

## Materials and Methods

### Diurnal variation of gas exchange collection

In 2003, 2-year-old *T. ramosissima* seedlings were transplanted in a uniform matrix in 2-m columns and 4-m rows in an ecological station located in Jingtai County (104.07E, 37.15N) at the southern edge of the Tenger Desert. None of the plants were watered after transplantation. The transpiration rate (*E*), vapour pressure deficit (VPD), leaf stomatal conductance (GH_2_O), net photosynthetic rate (*A*), intercellular CO_2_ concentration (*c*_i_), ambient CO_2_ concentration (*c*_a_), water out of the leaf chamber (*w*_o_) and water into the leaf chamber (*w*_i_) were measured in the field using a Walz GFS-3000 portable photosynthesis system (Heinz Walz GmbH, http://www.walz.com/). One assimilative branch was measured using a leaf area-specific adapter. The average value of the photosynthetic parameters from three assimilating branches was used for further analysis. The air temperature in the leaf chamber of the GFS-3000 was maintained at a constant 25 °C, the simulation light intensity was set at 1200 μmol·m^−2^·s^−1^ and the air flow was maintained at 750 mol·s^−1^. The CO_2_ concentrations and humidity were dependent on external conditions. Each sample was measured three times. Before data collection, water present on the surface of the leaves was wiped off with a tissue. Diurnal data were collected across a 24-h cycle for 2 days (18th and 28th July) with an average to go best curve, and individual points were eliminated according to variance.

### High air humidity exposure experiment

In the high moisture exposure experiment, it was hard to find plants in the natural field similar to those in the controlled chambers. Soil elements and root bifurcation could also complicate water conduction analysis in adult plants in field experiments. To eliminate these factors and to achieve consistency, we attempted to find one tree roughly divided into two main branches separating from the ground, with a crown width of 2.95 ± 0.05 m in the west–east direction and 3.25 ± 0.05 m in the north–south direction. One branch was exposed to natural conditions and served as the control, and the other branch that was exposed to a controlled humidity chamber was the treatment group. The humidity-controlled chamber occupied approximately 3 × 1.8 × 1.8 m^3^ with plexiglass sheets. Two ultrasonic humidifiers (Yadu Electronics, Beijing, China) were used to increase atmospheric moisture to 85 % in the chamber. Three thermohygrographs (MicroLog PRO-EC750; Fourier Systems Ltd, Israel) monitored the air temperature and RH every 5 min, and the values were calibrated with an automatic weather station (AWS TypeWS01; Delta-T Corp., UK).

### Gas exchange measurement

The *E*, VPD, GH_2_O, *A*, *c*_i_, *c*_a_, *w*_o_ and *w*_i_ were measured in the field using a Walz portable photosynthesis system (GFS-3000; Heinz Walz GmbH, http://www.walz.com/). One fully expanded leaf per individual was measured using the leaf area-specific adapter. The air temperature was kept constant (25 °C) in the leaf chamber of the GFS-3000, the simulation light intensity was set at 1200 μmol·m^−2^·s^−1^ and the air flow was maintained at 750 mol·s^−1^. The CO_2_ concentrations and humidity were dependent on external conditions. Each sample was measured three times. Before data collection, water present on the leaf surface was wiped off with tissues.

### Sap flow velocity

Heat-pulse sap flow sensors (SF100; Greenspan Technology, Coffs Harbour, Australia) on sap flow gauges (Flow32; Dynamax Inc., Houston, TX, USA) were used to continuously monitor sap velocity using the energy balance principle. Heat-pulse velocity was then converted into sap flow velocity using the algorithms developed by Edwards and Warwick (1984). All the calculations were integrated using the SAPCAL software. In the diurnal experiments, two vigorous branches, one each inside and outside the chamber, were separately implemented with three gauges set 20 cm apart from each other to obtain the integral sap flow velocity despite the diameter effect. Positive values for sap flow velocity were regarded as water storage depletion, while negative values were regarded as water replenishment or storage. Here, we used negative sap flow velocity as an indicator of the occurrence of FWU.

### Collection of leaves for gene expression studies

To identify the differently expressed genes between the humidity exposure and natural field conditions at 21:30 on 28 July 2013, a minimum of 5 g of fresh leaves were collected from at least three branches inside the plexiglass chamber (high humidity stress, HHS) and from leaves growing under natural conditions (Control, CT). All the collected leaves were placed into liquid nitrogen immediately after being collected from the tree, and the samples were transported to the laboratory and stored at −80 °C. Fresh leaf samples obtained from three branches of the same plants were used as three biological replicates. Total RNA from the three biological replicates was mixed to construct a cDNA library.

### RNA extraction, library preparation and RNA-seq

Total RNA was extracted using the EZNA® Plant RNA kit (Omega Bio-Tek, Norcross, GA, USA) with 2 % polyvinylpoly pyrrolidone. The remaining DNA was removed with RNase-free DNase (Omega Bio-Tek) according to the manufacturer’s instructions. Total RNA concentration and purity were assessed by optical density (OD) 260/280 nm ratios as determined using NanoDrop1000 (Thermo Scientific, Waltham, MA, USA). All the samples passed quality control analyses with OD 260/280 ratios ranging from 1.9 to 2.2 and OD 260/230 ratios ≤ 2.0. RNA integrity was verified by 1.5 % agarose gel electrophoresis with two distinct 28S/18S ribosomal RNA bands demonstrating good RNA integrity.

Total RNA (minimum 6 µg) was used to construct the DGE libraries using an Illumina gene expression kit (Illumina, Inc.; San Diego, CA, USA) according to the manufacturer’s protocol (version 2.1B). Poly(A) mRNA was enriched by magnetic oligo(dT) beads, and then interrupted into 200–700 nt (nucleotide) fragments. Using these short fragments as templates, the first cDNA strand was synthesized by random hexamer primers, followed by second-strand cDNA synthesis using DNA polymerase I (New England BioLabs) and RNase H (Invitrogen). The short DNA fragments were purified with a QiaQuick polymerase chain reaction (PCR) extraction kit (QIAGEN Inc., Valencia, CA, USA) for end-repairing and A-tailing. Then, the DNA fragments were ligated to sequencing adaptors, and DNA fragments of the required length were then purified by agarose gel electrophoresis and gathered by PCR amplification. Finally, a paired-end library was sequenced in an Illumina HiSeq™ 2000 sequencer with an average read length of 100 bp. Raw sequences were transformed into clean tags by filtering out adapter-only tags and low-quality tags. All the clean tags were then assembled into transcripts by Trinity package with default settings.

### Sequence annotation and identification of DEG pathways

The final assembled transcripts (≥100 bp) were submitted for homology and annotation searches using the Blast2GO software v2.4.4 (Conesa *et al.* 2005). All sequences were annotated by aligning with public protein and nucleotide databases. Four annotation databases, namely, the National Center for Biotechnology Information non-redundant protein database (Nr), the SwissProt protein database (SwissProt), the Clusters of Orthologous Groups of proteins category (COGs) and the Kyoto Encyclopaedia of Genes and Genomes (KEGG) database were used to validate and annotate the assembled genes with an *E*-value cut-off of 1e-5. Based on the alignment results, further annotation analysis was performed using gene ontology (GO) terms describing biological processes, molecular functions and cellular components using the Blast2GO software. The number of reads per kilobase per million mapped reads (RPKM) of each unigene was normalized by the ERANGE 3.1 software to determine the unigene expression profiles (Mortazavi *et al.* 2008). The false discovery rate (FDR) ranking was used to adjust the *P-*value in multiple tests and analyses (Qin *et al.* 2011). Transcripts with a difference of at least 2-fold (absolute values of log_2_ (Ratio) ≥ 1 with FDR < 0.001) were regarded to have significant differential expression.

## Results

### Validation of FWU by sap flow velocity in *T. ramosissima*

Obviously, there was reverse sap flow on both days. Slightly negative sap flow occurred at about 20:30 and reached a minimum at about 22:00, then slowly increased. The pattern and amount of sap flow velocity were similar to those on 28th during the daytime. The sap flow velocity was higher at night on the 18th than on the 28th. After analysing the meteorological factors, we found that the time lag shift and small sap flow velocity were attributed to the slight rain during the daytime on the 18th to maximize photosynthesis, which suggested that the drier environment resulted in earlier absorption on 28th, and ampler moisture supply resulted in greater negative sap flow velocity. After humidity exposure (<85 % humidity), the sap flow velocity tended to have a lower negative value. The pattern of sap flow velocity under humidity exposure was quite different from that under the control conditions. With the increase of humidity by humidifier, the amount of sap flow velocity sharply decreased to a minimum at 21:48 on 28th, which might be due to the high air humidity caused by the humidifier.

### Diurnal characteristics of photosynthetic variation under the humidity condition

Under natural field conditions, the photosynthetic rate was bimodal with a midday depression, and other photosynthetic parameters were consistent with the characteristics of C_3_ plants (Fig. 2). Under natural field conditions, there was a short-term lag in plant adaptation when environmental factors changed from one state to another. Although the environmental factors changed a lot in a day, their impact on plants was gentle or stable in a short period of time. However, when the saturated water vapour difference reached a certain degree, the influence on the plants’ stomata was large enough to result in significant changes in photosynthetic parameters. The *E*, *A*, VPD and *c*_a_ were lower in the daytime, whereas GH_2_O, *w*_o_ and *c*_i_ were higher at night under natural field conditions than those in the presence of high humidity.

**Figure 1.**
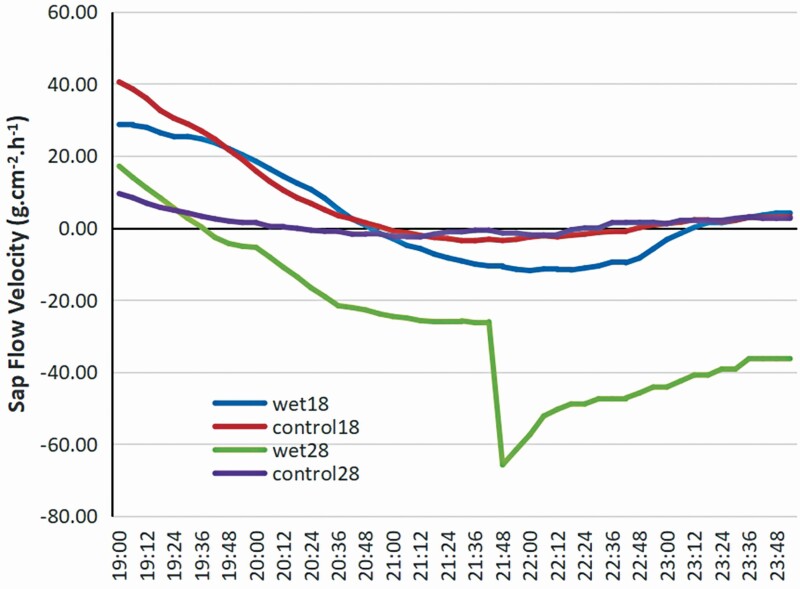
Diurnal variation of sap flow velocity on 18th and 28th July. The yellow and red lines represent sap flow velocity (g·cm^−2^·h^−1^) under natural conditions on 18th and 28th July, respectively. The blue and grey lines represent sap flow velocity under the wetting condition with humidifier on 18th and 28th July, respectively.

**Figure 2.**
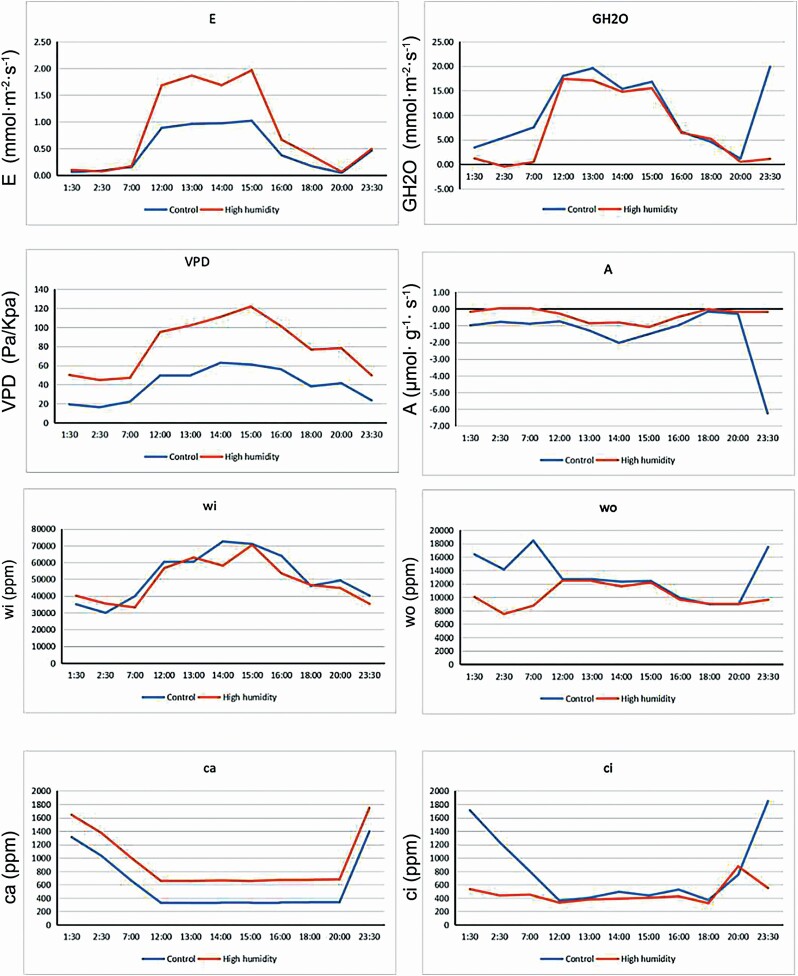
Comparison of diurnal photosynthetic variation between natural field and humidity conditions. *E*, transpiration rate; VPD, vapour pressure deficit; GH_2_O, leaf stomatal conductance; *A*, net photosynthetic rate; *c*_i_, intercellular CO_2_ concentration; *c*_a_, ambient CO_2_ concentration; *w*_o_, water out leaf chamber (ppm); *w*_i_, water into leaf chamber.

To determine the inconsistencies in the GH_2_O, *w*_o_ and *w*_i_ values between day and night, we performed the humidification experiment. From Table 1, the photosynthetic parameters, except VPD and *c*_i_, between the humidity and natural field conditions were highly correlated until the moisture content in the air reached 85 %. That is to say, the effects of water absorption on photosynthesis and respiration were very small at night. On the contrary, the VPD, *A* and *w*_o_ values were highly correlated when the humidity exceeded 85 %. This change in the correlation between parameters reveals a fundamental shift in the pathway of carbon assimilation, while the respiration pattern did not change much apart from quantitative variations.

**Table 1.** Relationship of photosynthetic parameters under controlled humidity and natural field conditions.

Parameters	Lower than 85 %		Higher than 85 %	
	PCC*	*P*-value	PCC*	*P*-value
*E*	0.98	0.02	0.39	0.03
VPD	0.75	0.06	0.71	0.01
GH_2_O	0.99	0.04	0.35	0.02
*A*	0.89	0.01	0.64	0.04
*c*_i_	0.95	0.08	0.03	0.01
*c*_a_	0.98	0.03	−0.21	0.01
*w*_o_	0.99	0.02	0.63	0.00
*w*_i_	0.80	0.05	0.48	0.49

*PCC, Pearson correlation coefficient.

As shown in Table 2, there was a significant decrease in four parameters (namely, *E*, VPD, GH_2_O and *w*_i_ with variations of 63 %, 11 %, 57 % and 9 %, respectively), suggesting that there was a low transpiration rate under high humidity conditions at night. The increased percentages of *A* and *c*_i_ (40 % and 17 %, respectively) indicated that carbon accumulation occurred under conditions of high humidity at night. The changes in *c*_a_ and *w*_a_ were very small (0.04 % and 0.14 %, respectively), suggesting that the change in gas had very low relevance with the extracellular environment. The positive *c*_i_, *A* and negative GH_2_O levels suggested that stomata were not the sole channels by which water entered leaves. Thus, we believe that the specialized structures, such as salt glands or cuticles, could play a significant role in water movement across the leaf surface in *T. ramosissima*. The results suggested that plants absorbed the water on the leaf surface on the one hand, and reduced transpiration to keep the water absorbed and increased photosynthesis to fix the water under conditions of high air humidity on the other hand.

**Table 2.** Comparison of photosynthesis parameters between control and wet leaves.

Parameters	Control (20 %)	Wet (85 %)	Variation
*E*	0.05	0.02	−63 %
VPD	41.46	36.73	−11 %
GH_2_O	1.14	0.49	−57 %
*A*	−0.29	−0.18	40 %
*c*_i_	747.63	872.51	17 %
*c*_a_	340.28	340.40	0 %
*w*_a_	9012.40	9000.01	0 %
*w*_i_	49 267.74	44 744.40	−9 %

### Global transcriptome profiling for genes response to moisture

To obtain a global view of differential gene expression at the transcriptional level in *T. ramosissima* under conditions of high humidity at night, a pooled cDNA library of leaves was constructed under both natural field (CT) and high humidity conditions (HHS). A data set with a total size of 4.8 gigabase was generated from 120 585 836 clean paired-end reads with a mean length of 108 bp and a Q20 of over 99 % (Table 3). The number of unique mapped transcripts was 6 713 731 (25.47 %) and 6 987 269 (25.88 %) in RNA12 (CT) and RNA13 (HHS) samples, respectively. Combined with multi-mapped transcripts, the total mapped transcripts accounted for 91.87 % and 91.59 % in CT and HHS samples, respectively. A total of 72 035 unigenes were annotated, of which 29 683 (41.21 %) were from the Nr database, 8818 (12.24 %) were from the KEGG database, 19 665 (27.30 %) were from the SwissProt database and 18 863 (26.18 %) were from the COGs (**see****Supporting Information—Figure**). Despite the short length of the transcripts, there were still 42 211 (58.60 %) unigenes unannotated in *T. ramosissima*. Table 3 lists the details of sequencing data of the DEG. The sequence output and quality were adequate for further analysis.

**Table 3.** Statistics of DEG sequencing.

Sample	CT	HHS
Filtered bases (%)	5 320 688 832 (88.39 %)	5 449 978 036 (88.48 %)
Filtered reads (%)	52 725 714 (88.46 %)	54 001 096 (88.55 %)
Q20	99.44 %	99.45 %
Total reads	26 362 857	27 000 548
Unmapped reads	2 144 288 (8.13 %)	2 270 826 (8.41 %)
Uniq mapped	6 713 731 (25.47 %)	6 987 269 (25.88 %)
Multi-mapped	17 504 838 (66.40 %)	17 742 453 (65.71 %)
Total mapped	91.87 %	91.59 %

### Differently expressed unigenes in conditions of high humidity

**Figure 3.**
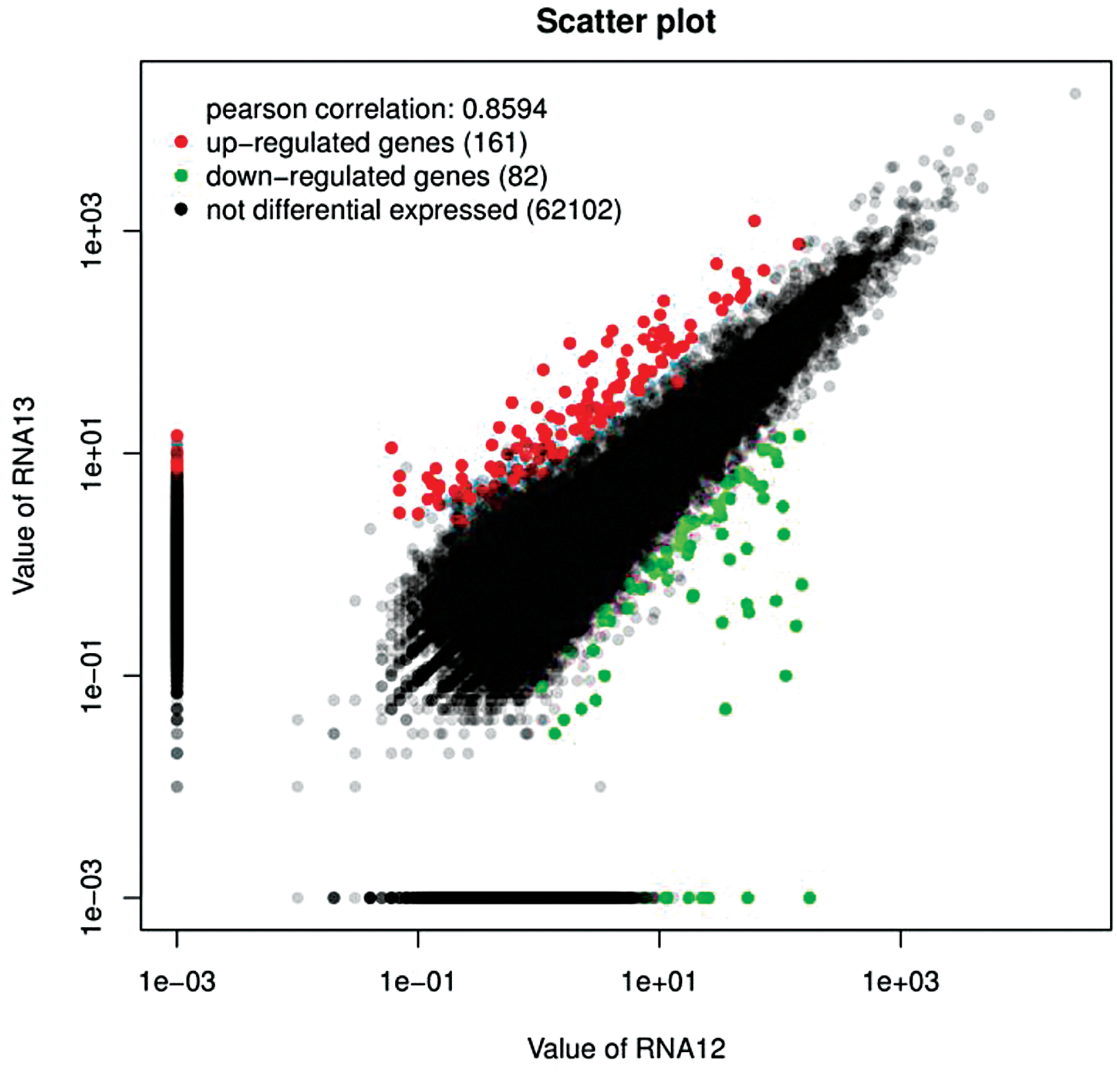
Comparison of gene expression profiles between the HHS and CT samples. Absolute values of log_2_ fold-change ≥ 1 and FDR ≤ 0.05 were used to judge the significance of DEGs. The red dots represent upregulated unigenes, and the green dots represent downregulated unigenes. The black portion represents unigenes that were not differentially expressed.

To compare differential expression patterns in conditions of high humidity, we normalized the distribution of transcripts for gene expression level in each library to first make an effective library size of log_2_ fold-change ≥ 1 by edgeR (Empirical analysis of Digital Gene Expression in R). Of the 73 025 unigenes, most of the unigenes displayed no differential expression between the HHS and CT groups (62 102, 86.21 %). There were 28 231 unigenes with more than 2-fold difference in abundance (either higher or lower) which was controlled with by the Fisher’s exact test. This number decreased to 2951 with FDR ranking (details shown in Fig. 3). Of all the 243 differential expressed genes (DEGs), 161 were upregulated and 82 were downregulated in HHS samples.

### GO and COG classification

**Figure 4.**
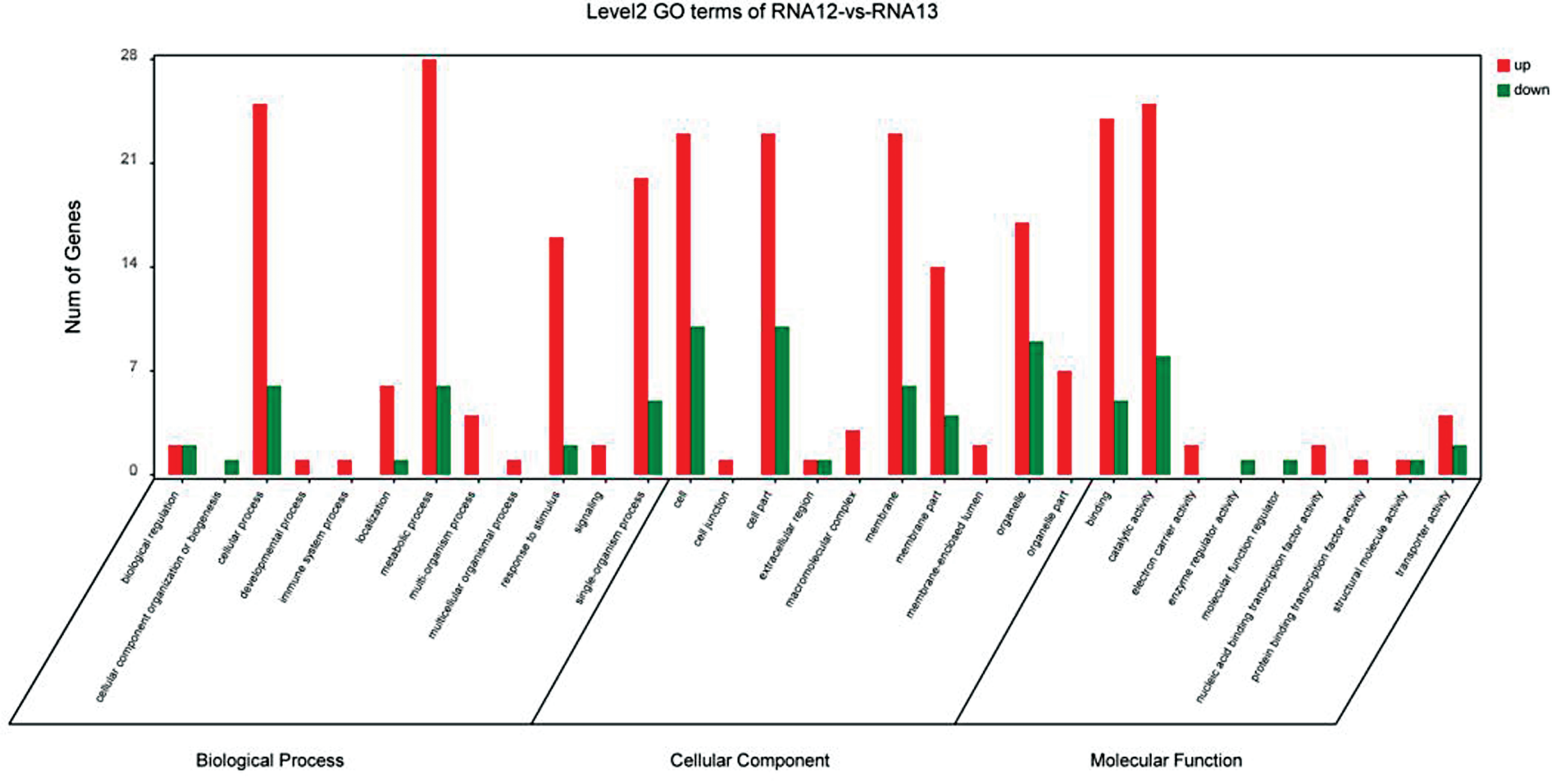
Gene ontology categories of the identified DEGs.

Gene ontology assignments were used to classify the functions of DEGs with at least 2-fold changes in expression (Fig. 4). The DEGs were then classified into unique GO functional terms of biological process (54 unigenes, 22.22 %), cellular component (52 unigenes, 21.40 %) and molecular function (49 unigenes, 20.16 %). In terms of biological process, most of the unigenes were clustered into ‘metabolic process’ (34 unigenes, 14 %), ‘cellular process’ (31, 12.76 %), ‘single-organism process’ (25, 10.29 %) and ‘response to stimulus’ (18, 7.41 %). In terms of cellular components, most of the unigenes were clustered into cells (33, 13.58 %), (32, 13.17 %), membranes (29, 11.93 %) and organelles (26, 10.70 %). In terms of molecular function, 49 of 243 genes were clustered into ‘catalytic activity’ (33, 13.58 %), ‘binding’ (29, 11.93 %) and ‘oxidoreductase activity’ (12, 4.94 %).

Table 4 lists the DEGs with significant GO annotations. Among the genes associated with carbohydrate metabolism, Unigene025103 (acidic endochitinase-like), Unigene034393 (chitotriosidase-1-like), Unigene035483 (chitinase 3), Unigene 070115 (basic endochitinase CHB4-like) and Unigene031580 (6-phosphofructokinase 3-like) were upregulated to degrade starch and promote the glycolytic process, which ultimately facilitates pyruvate production and dehydrogenation to acetyl-CoA. Downregulation of Unigene027016 (probable pectate lyase 5) decreased the cellulose, hemicellulose and soluble sugar contents, which can provide enough activated acetic acid and is present in the form of acetyl-CoA in Tricarboxylic Acid Cycle (TCA) circulation, accompanied with energy transfer. For genes involved in energy transfer, the hydrogen carriers (NADH + H and FADH2) generated in the circulation will release more energy in the cell respiratory chain. In our study, most of the DEGs involved in energy transfer were upregulated following exposure to high humidity, including Unigene001618 (cytochrome *c* oxidase subunit I), Unigene009205 (apocytochrome *b*), Unigene011021 (cytochrome *c* oxidase subunit I), Unigene038856 (ATP synthase subunit alpha), Unigene058214 (ATPase subunit 9), Unigene058513 (cytochrome *c* oxidase subunit 3), Unigene061258 (cytochrome *c* oxidase subunit 3), Unigene063584 (NADH dehydrogenase subunit 4), Unigene063908 (cytochrome *c* oxidase subunit II), Unigene069302 (ATPase F0 subunit 6) and Unigene070977 (ATP synthase subunit beta). On the other hand, Unigene038863 (ATP synthase subunit alpha) was downregulated to promote respiration and to increase the intercellular CO_2_ concentration. In other words, the plants prioritized survival over growth in the drought environment. For pyruvate metabolism, the pyruvate dehydrogenase complex (Unigene032200) catalyses the overall conversion of pyruvate to acetyl-CoA and CO; malate dehydrogenase (MDH; Unigene024210) catalyses a reversible NAD-dependent dehydrogenase reaction involved in central metabolism and redox homeostasis between organelle compartments, and plays an essential role in autotrophic metabolism in photosynthetic tissues. In terms of transmission of genetic information, apurinic endonuclease-redox protein and ribosomal protein S10 were downregulated to repress DNA damage repair. Heterogeneous nuclear ribonucleoprotein 1-like (Unigene016965) and 6-phosphofructokinase 3-like (Unigene031580) might integrate different signalling pathways to direct RNA transcription and protein translation, folding, sorting and degradation, and most importantly, the phosphatidylinositol signalling system (Unigene000853, Unigene000854 and Unigene011919) and plant hormone signal transduction (Unigene013040, Unigene017342, Unigene031201 and Unigene058078).

**Table 4.** DEGs with significant GO annotations.

Unigenes	log_2_(fold-change)	Annotation
Carbohydrate metabolism		
Unigene025103	2.63	Acidic endochitinase-like
Unigene034393	4.77	Chitotriosidase-1-like
Unigene035483	2.56	Chitinase 3
Unigene070115	13.00	Basic endochitinase CHB4-like
Unigene031580	2.84	6-Phosphofructokinase 3-like
Unigene027016	−3.64	Pectate lyase 5
Genes involved in energy transfer		
Unigene001618	3.96	Cytochrome *c* oxidase subunit I
Unigene063908	3.73	Cytochrome *c* oxidase subunit II
Unigene061258	11.68	Cytochrome *c* oxidase subunit 3
Unigene011021	3.61	Cytochrome *c* oxidase subunit I
Unigene009205	3.51	Apocytochrome *b*
Unigene038856	−6.79	ATP synthase subunit alpha
Unigene069302	13.82	ATPase F0 subunit 6
Unigene058214	3.57	ATPase subunit 9
Unigene070977	4.84	ATP synthase subunit beta
Unigene063584	12.83	NADH dehydrogenase subunit 4
Pyruvate metabolism		
Unigene012360	−2.08	Pyruvate kinase
Unigene014495	−2.11	Acetyl-CoA carboxylase carboxyltransferase subunit alpha
Unigene019849	1.49	Acetyl-CoA carboxylase carboxyltransferase beta subunit
Unigene037464	2.15	Aldehyde dehydrogenase family 3 member I1
Unigene024210	1.10	Malate dehydrogenase
Unigene028831	−9.25	Acetyl-CoA synthetase, chloroplastic/glyoxysomal-like
Unigene032200	1.49	dihydrolipoyllysine-residue acetyltransferase component 2 of pyruvate dehydrogenase
Unigene040553	−1.21	Phosphoenolpyruvate carboxylase 2
Phosphatidylinositol signalling and plant hormone signal transduction		
Unigene000853	11.76	Calmodulin
Unigene000854	3.75	Calmodulin
Unigene011919	12.06	Calmodulin
Unigene013040	3.16	Auxin-induced protein X15
Unigene017342	3.38	Pathogenesis-related leaf protein 6-like
Unigene058078	2.55	Basic helix-loop-helix family protein
Reference genes		
Unigene054230	−2.82	Apurinic endonuclease-redox protein
Unigene016965	5.38	Heterogeneous nuclear ribonucleoprotein 1-like
Unigene052585	−5.17	Ribosomal protein S10
Unigene069446	4.87	Ubiquitin
Unigene031580	2.84	6-Phosphofructokinase 3-like
Unigene047544	4.31	Elongation factor-1 alpha, partial
Unigene025927	3.02	Heat shock protein 70
Plant–pathogen interaction		
Unigene011919	12.06	Calmodulin
Unigene017342	3.38	Pathogenesis-related leaf protein 6-like
Unigene021895	−10.61	Cyclic nucleotide-gated ion channel 5 isoform X2
Unigene030777	3.53	Calcium-binding protein CML44

Genes involved in both pathways were upregulated, which increased the adaptation of plants to an extreme drought environment. Interestingly, some of the genes that are often considered to be less affected by environmental factors were found to be highly upregulated by humidity stress, such as ubiquitin (Unigene069446, 4.87), elongation factor-1 alpha (Unigene047544, 4.31) and *HSP70* (Unigene025927, 3.02), suggesting that these processes were universal during FWU. For plant–pathogen interaction, the gene encoding pathogenesis-related leaf protein (Unigene017342) was upregulated to affect leaf physiology and potentially water movement. All those signalling were highly correlated with phosphatidylinositol signalling and plant hormone signal transduction (Unigene000853, Unigene000854, Unigene011919, Unigene013040 and Unigene058078), and facilitate downstream transmission of the cascade signal.

**Figure 5.**
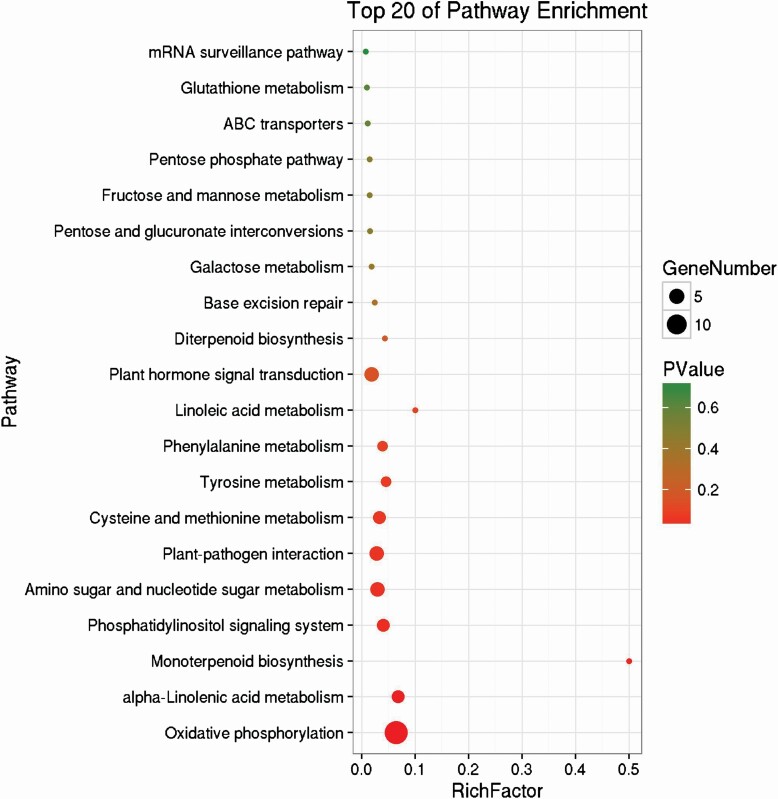
The most significantly enriched KEGG pathways. The size of the circle represents gene numbers in each pathway, the colour from red to green represents the *P-*value for each pathway.

To further gain insights into the biological functions of and interactions among our identified unigenes, a pathway analysis based on the roles in biochemical pathways was performed in the KEGG pathway database. Of all the 27 enriched KEGG pathways, the following eight had the most significant enrichments, including ‘oxidative phosphorylation’, ‘alpha-linolenic acid metabolism’, ‘monoterpenoid biosynthesis’, ‘phosphatidylinositol signalling system’, ‘amino sugar and nucleotide sugar metabolism’ and ‘plant–pathogen interaction’. Figure 5 presents a list of the top 20 enriched KEGG pathways and their significance.

**Figure 6.**
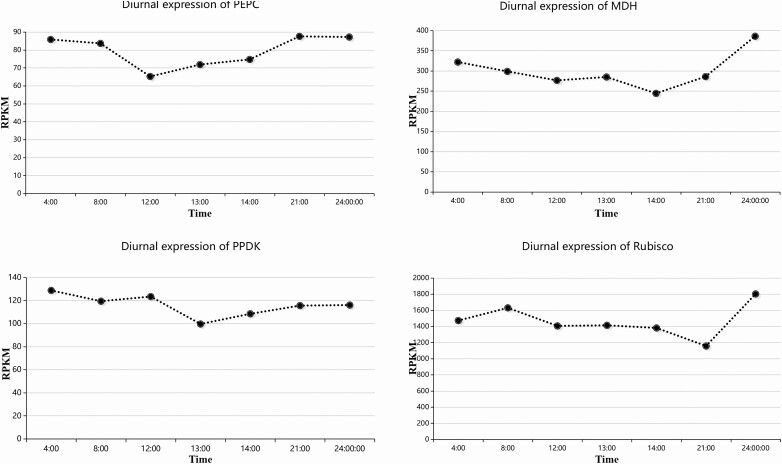
Diurnal expression of key genes involved in carbon assimilation. A, Diurnal expression of PEPC; B, Diurnal expression of MDH; C, Diurnal expression of PPDK; D, Diurnal expression of Rubisco.

**Figure 7.**
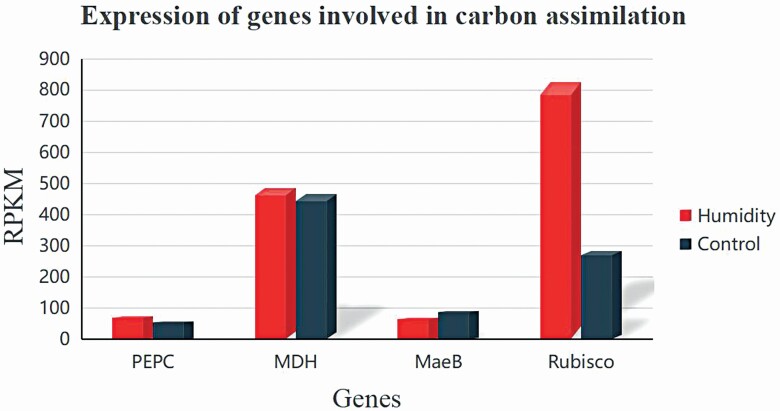
Expression of key genes involved in carbon assimilation under conditions of high humidity.

To investigate diurnal variations and the differential expression of the key genes of the three carbon assimilation types after growing the plants in a humidified atmosphere, diurnal expression patterns of four typical genes were further analysed based on the transcriptome data (unpublished data). As shown in Fig. 6, a decrease in the PEPC occurred in the morning and PEPC was maintained at a low level until 21:00, suggesting that PEPC was repressed in the daytime and activated at night (Fig. 6A). Consistent with this finding, MDH remained low in the daytime and increased sharply at night (Fig. 6B). The expression levels of PEPC and MDH were highly correlated with that of the AirRh (PCC was 0.66 and 0.63, respectively, and the *P-*values in both cases were <0.01). *MaeB* displayed a slight variation except at noon (Fig. 6C), which may have contributed to the photosynthetic noon break. Rubisco peaked in the morning, and remained high during both the day and night (Fig. 6D). Except for *MaeB*, all the other three genes were upregulated by the humidity over 85 % (Fig. 7).

## Discussion

### Physical and biological characteristics of FWU

Many studies have shown that the amount of water from foliar absorption increases with the aggregation of drought. In the coastal prairie ecosystem of California, 28–66 % of the water intake relies on FWU (Corbin *et al.* 2005). This percentage increases to 34 % in the redwood tree ecosystem (Dawson and Goldsmith 2018) and reaches 74 % in the Negev desert, Israel (Kidron 2010). According to these observations, drought seems to be a prerequisite for leaf water absorption. To clarify the adaptation mechanism of FWU in *T. ramosissima*, we used sap flow, diurnal meteorological data and photosynthesis parameters to determine the physical and biological characteristics of FWU.

Foliar water uptake has often been observed when the air is saturated with moisture and liquid water forms on leaves. The sap flow pattern of the control group at night on 18 July was similar to that on 28 July, then gradually decreased with the passage of time due to increase in the air humidity (Fig. 1), whereas the sap flow plummeted to an extremely low value at approximately 21:30 on 28 July. Combined with the VPD (from 41.46 to 36.73) and the *w*_i_ value (from 49 267 to 44 744), the phenomenon may contribute to the resistance of a phase change from vapour to liquid. Due to the magnitude of the gauges of the device, there is also evidence of FWU of water vapour when the air has not been condensed into liquid water but there have been no studies that have quantified these fluxes experimentally (Berry *et al.* 2019). However, theoretically, if the conductance of the leaf surface (cuticle or stomata) does not vary, more negative leaf water potentials should lead to higher FWU fluxes (Limm *et al.* 2009; Goldsmith *et al.* 2013). Vapour uptake would be driven by vapour pressure and require the intercellular air space immediately within the leaf cuticle to have a lower vapour pressure than that of air. This routinely occurs in leaf air spaces due to negative water potentials and the Kelvin effect reducing the vapour pressure (Cernusak *et al.* 2018). Thus, any movement from intercellular air space into cells would require liquid water, and the resistance of a phase change from vapour to liquid should be regarded. Thus, the FWU is driven by the vapour concentration gradient instead of water potentials, which are impacted by many factors, including RH and leaf temperature. For instance, at 25 °C, the water potential of the air drops below −4 MPa at 0.036 kPa VPD (97 % RH) (Berry *et al.* 2019). This may result in the shift of CO_2_ assimilation, stomatal conductance and transpiration rate (Berry *et al.* 2019) with significant variations in pore size and stomatal density in conditions with humidity of up to 85 % (Kim *et al.* 2018). Reliable tracking equipment should be developed to monitor the movement of individual water molecules from FWU, which would open doors to explaining many phenomena in the process of FWU.

### Balance between energy demand and respiration after FWU

Genes with increased expression at night and decreased expression during the day may be important for water storage and carbon fixation (Zhang *et al.* 2019). To have a global view of responsive genes in the process of FWU in *T. ramosissima,* we constructed a cDNA library of leaves obtained from a humidification experiment. A total of 161 unigenes were upregulated to increase FWU and carbon fixation, and 82 were downregulated to decrease the water and carbon loss (Fig. 3). The great number of genes involved in ‘oxidative phosphorylation’ reveals the balance between energy demand and respiration. For example, the unigenes encoding NADH dehydrogenase (Unigene063584), cytochrome *C* (Unigene058513), and oxidase (Unigene009205) were downregulated to produce fewer protons, and ATP synthase (Unigene038856, Unigene038863 and Unigene070977) with different binding change mechanisms to retain H^+^ (Table 1). Other KEGG pathways, including ‘alpha-linolenic acid metabolism’, ‘monoterpenoid biosynthesis’, ‘phosphatidylinositol signalling system’, ‘amino sugar and nucleotide sugar metabolism’, ‘plant–pathogen interaction’, ‘cysteine and methionine metabolism’ and ‘tyrosine metabolism’ were among the most significantly enriched categories, and these pathways are indispensable for carbon assimilation (Fig. 5). These findings suggested that carbon metabolism was highly related to the FWU at night.

### A combination of C_3_ and CAM carbon assimilation in *T. ramosissima* under high humidity conditions in the desert

*Tamarixramosissima* has long been regarded as a C_3_ plant. However, all the core genes of C_4_ carbon fixation were found not only in the two *Tamarix* species *T. chinensis* and *T. ramosissima* (Swaminathan *et al.* 2020), but also in another species in the same family: *R. soongorica* (Shi *et al.* 2013). Thus, *Tamarix* species have the molecular basis to utilize C_4_ carbon fixation together with the C_3_ pathway.

Diurnal expression patterns (Fig. 6) showed that the expression of the typical C_3_ metabolism Rubisco gene peaked in the morning and remained high during the daytime and night with a typical sap flow velocity photosynthesis curve. Meanwhile, essential C_4_-photosynthetic genes such as *PEPC* and *MaeB* displayed a photosynthetic noon break. These results suggested that the fate of CO_2_ assimilated at night limited CAM activity when the humidity was low. Considering that the core genes of C_3_ and C_4_ metabolism were upregulated under high moisture exposure, we agreed with the viewpoint that the plant could use starch to maximize its carbon absorption at night (Smith and Zeeman 2020). Thus, like many plant species, *T. ramosissima* exhibits facultative CAM, switching between CAM and C_3_ photosynthesis depending on drought and other abiotic conditions (Bangal and Lambrecht 2010). The combination of C_3_ and CAM metabolism in *T. ramosissima* could be a common pattern for desert plants to help them survive in a harsh environment.

This study may provide not only an important theoretical foundation for the engineering of high-yield drought-tolerant plants and the conversion of C_3_ plants to CAM plants, but also may broaden the knowledge of plant water physiology and restoration of desert plants in arid regions. Nevertheless, there is a long way to go to understand the strategy adopted by desert plants to survive. For example, it is easier to activate a PEPC pathway than to study leaf anatomy (Arantes *et al.* 2020). Even if the CAM pathway does not require functional and anatomic specialization, water transport and storage remain very complex processes. Further experiments should be performed to validate and strengthen the current conclusions.

## Conclusions

Foliar water uptake can improve plant–water relations and lead to an increase in the plant’s performance in response to the environment. We used DEGs to globally explain the molecular mechanism of FWU and reported the combination of C_3_ and CAM metabolism pathways utilized by desert plants to survive in a harsh environment. However, many questions regarding the pathways and the implications of FWU remain unanswered, such as the environmental impact and gene evolution. One clear theme is that FWU not only plays an important role in plants but it may also be an important tool for plants to mitigate climate change-driven drought in the future.

## Supporting Information

The following additional information is available in the online version of this article—

**File 1.** Four gene expression of carbon assimilation.

**File 2.** Meteorological variation of three days.

**File 3.** Sap flow velocity in two days.

**File 4.** RNA12-vs-RNA13.genes.

**File 5.** RNA12-vs-RNA13.genes.filter.

**Figure 1.** RNA12-vs-RNA13.DE.scatter.

**Figure 2.** RNA12-vs-RNA13.DE.volcano.

**Figure 3.** KOG function classification of plant sequence.

plab060_suppl_Supplementary_Materials

## Data Availability

The data collected in this study are available as **Supporting Information**.

## Notes

**Plants, Ecosystems & Climate. Chief Editor**: Mary Heskel
