# Supplementary figures and images for: Transcriptome profiling reveals that foliar water uptake occurs with C3 and crassulacean acid metabolism facultative photosynthesis in Tamarix ramosissima under extreme drought
Source: AoB Plants. 2022 Jan 17;14(1):plab060. doi: 10.1093/aobpla/plab060 (PMC8763614; doi:10.1093/aobpla/plab060)

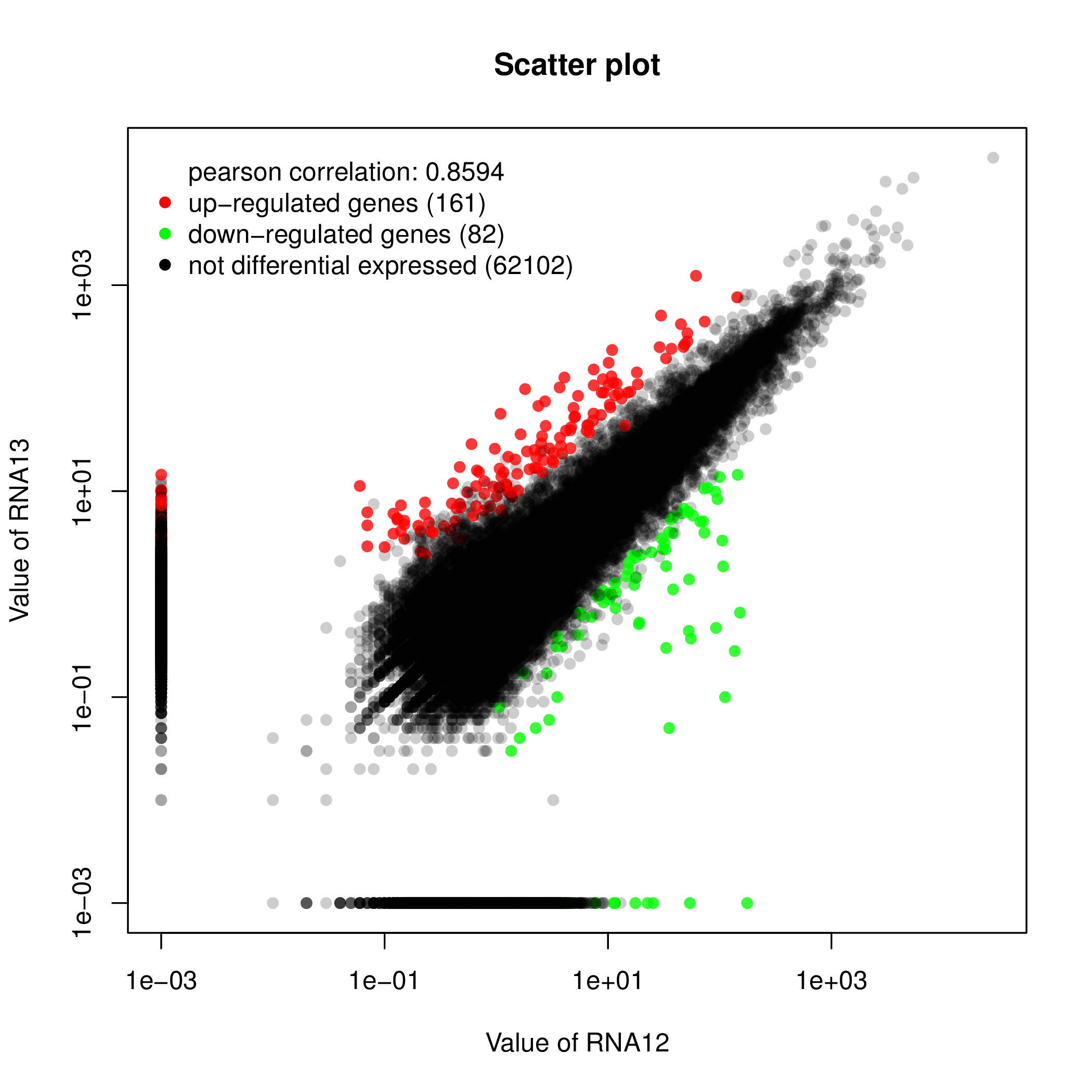

Supplement: plab060_suppl_Supplementary_Materials [file plab060_suppl_supplementary_materials.zip › plab060_suppl_RNA12-vs-RNA13.DE.scatter.png]

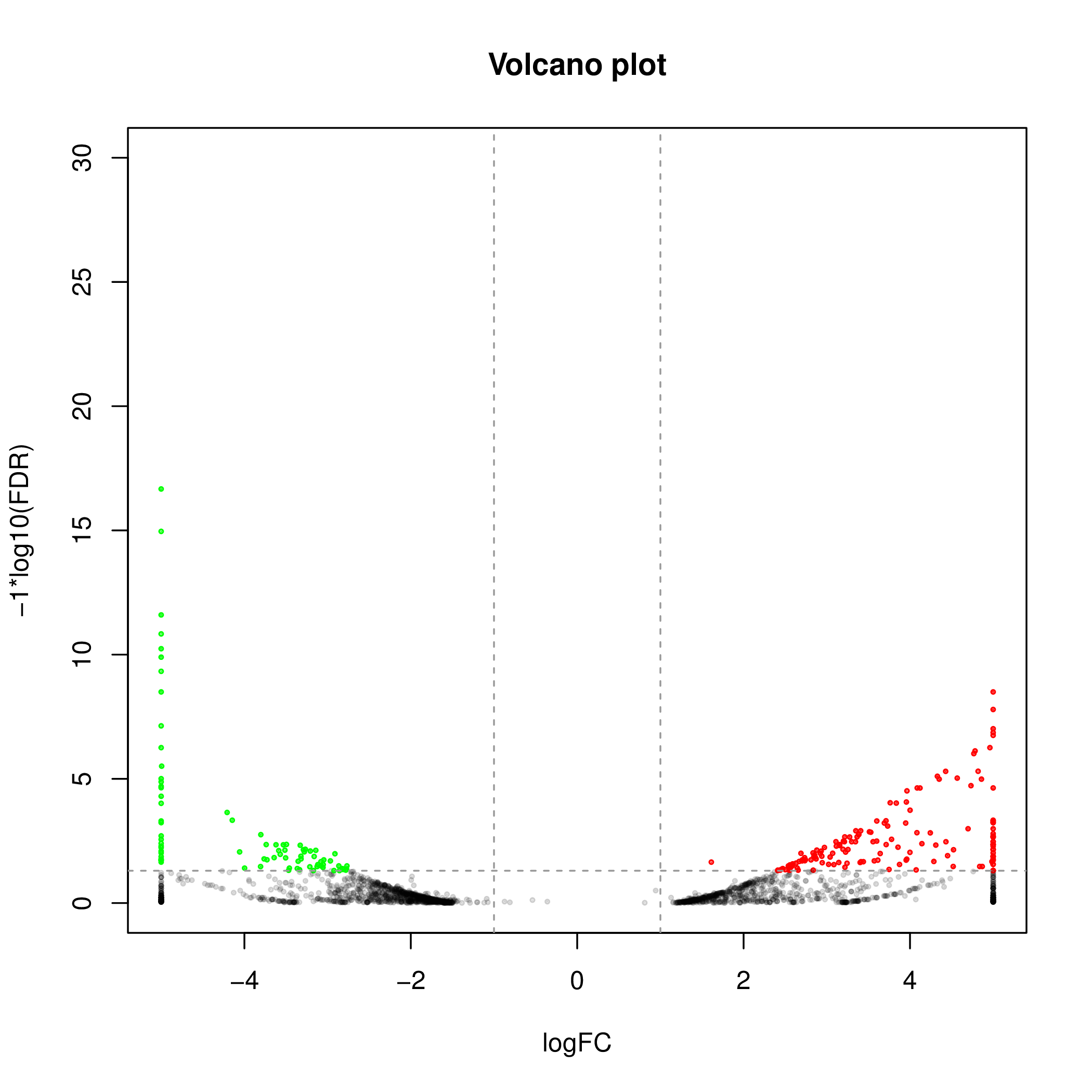

Supplement: plab060_suppl_Supplementary_Materials [file plab060_suppl_supplementary_materials.zip › plab060_suppl_RNA12-vs-RNA13.DE.volcano.png]
